# Evaluation of a Vaccine Candidate Designed for Broad-Spectrum Protection against Type A Foot-and-Mouth Disease in Asia

**DOI:** 10.3390/vaccines12010064

**Published:** 2024-01-09

**Authors:** Sung Ho Shin, Seong Yun Hwang, Hyun-Mi Kim, Se Hee Shin, Mi-Kyeong Ko, Min Ja Lee, Su-Mi Kim, Jong-Hyeon Park

**Affiliations:** 1 Center for Foot-and-Mouth Disease Vaccine Research, Animal and Plant Quarantine Agency, 177 Hyeoksin 8-ro, Gimcheon City 39660, Gyeongsangbuk-do, Republic of Korea; imshin121@korea.kr (S.H.S.); hsy8592@korea.kr (S.Y.H.); khm852456@korea.kr (H.-M.K.); mkk80@korea.kr (M.-K.K.); herb12@korea.kr (M.J.L.); beliefsk@korea.kr (S.-M.K.); 2 VAXDIGM, Room 335, 3rd Floor, 11, Bongeunsa-ro 63-gil, Gangnam-gu, Seoul 060097, Republic of Korea; ssh9147@vaxdigm.com

**Keywords:** foot-and-mouth disease, pig, vaccine, serotype A, broad protection

## Abstract

Foot-and-mouth disease (FMD) vaccines are currently the most powerful protective and preventive measures used to control FMD. In this study, the chimeric vaccine strain containing antigenic epitopes from the FMD virus serotype A, which belongs to the ASIA topotype, was produced and evaluated. The chimeric vaccine strains contain sea-97/G1 (VP4, VP2, VP3) and A22 Iraq (VP1) or G-VII (VP1) for use in FMD vaccines in Asia. The 50% protective dose was determined in mice. Vaccinated mice were challenged with three different type A viruses (Sea-97/G1, Sea-97/G2, G-VII clade) seven days post-vaccination (dpv), and mice that received the vaccine candidates were protected against the three viruses. The protective capability of one of the vaccine candidates was evaluated in pigs. Vaccinated pigs were challenged with three different type A viruses (Sea-97/G1, Sea-97/G2, G-VII clade) at 28 dpv, and pigs that received the vaccine candidate were protected against the three viruses. The results showed that this vaccine candidate, which was designed to provide protection against FMD in Asia, efficiently protected pigs against virus challenge and thus has potential as a broad-spectrum vaccine for various epidemic FMD viruses.

## 1. Introduction

Foot-and-mouth disease (FMD) is highly contagious and affects artiodactyl animals, such as cattle, sheep, deer, and pigs. There are seven FMD virus (FMDV) serotypes (SAT1, SAT2, SAT3, O, A, C, and Asia 1), and there is no cross-immunity among the serotypes [1]. Serotypes O, A, and Asia 1 continue to occur in Asia, which belong to pool 1. Among them, serotypes O and A mainly occur in East Asia.

Serotype A viruses are known to be the most diverse and genetically variable FMDVs, and there is imperfect cross-protection among them [2,3]. There have been three outbreaks of serotype A FMDV in the Republic of Korea since 2010. The type A FMDV that occurred in 2010 was of the Asia/Sea-97/G1 clade. This was followed by outbreaks in cattle in 2017 and in pigs in 2018 of viruses belonging to the Asia 1/Sea-97/G2 clade [4].

Since 2015, a novel type A virus, ASIA/G-VII, has been circulating after emerging from the Indian subcontinent [5], and a vaccine based on the A22 IRQ, A-SAU-95, and A-IRN-05 strains has been found to be ineffective against this novel virus [6]. This finding supports the notion that new vaccines for type A FMDVs generally need to be developed every 5 to 10 years because antigenically distinct subtypes emerge periodically [7].

The aim of this study was to develop a chimeric vaccine strain that can protect against the A/ASIA/Sea-97/G1 virus that occurred in the Republic of Korea in 2010, the A/ASIA/Sea-97/G2 virus that occurred in 2017 and 2018, and the A/ASIA/G-VII virus that is prevalent on the Indian subcontinent and difficult to prevent.

## 2. Materials and Methods

### 2.1. Cells and Viruses

Fetal goat tongue (ZZ-R 127) cells were passaged using Dulbecco’s modified Eagle’s medium (DMEM)/F12 (Corning, Union City, NJ, USA). Baby hamster kidney (BHK-21) cells and porcine kidney (LFBK) cells, supplied by the Plum Island Animal Disease Centro (Orient, NY, USA), were passaged using DMEM (Corning, Union City, NJ, USA) supplemented with 10% fetal bovine serums (Gibco, Paisley, Renfrewshire, UK), supplemented with 1% Antibiotic-Antimycotic (15240062; Gibco, Paisley, Renfrewshire, UK) in a 5% CO_2_ atmosphere incubator. Suspended BHK-21 cells were passaged using CD BHK-21 Production Medium (Gibco, Paisley, Renfrewshire, UK) added with sodium bicarbonate (S5761; Sigma-Aldrich, St. Louis, MO, USA) in a 5% CO_2_ atmosphere shaking incubator. The target virus sequences selected for this study were O_1_Manisa/Turkey/69 (O_1_manisa, GenBank No. AY593823.1), A/Pocheon/001/KOR/2010 (A/POC/2010, GenBank No. KC588943.1), A22 Iraq/24/64 (A22 Iraq, GenBank No. AY593763.1), A/BAN GA Sa-197/2013 (A/BAN/2013, GenBank No. KJ754939.1), and A/ASIA/NEP/12/2017 (A/NEP/2017, GenBank No. unregistered).

### 2.2. Site-Directed Mutagenesis and Subcloning

The chimeric viruses were obtained using the same procedures as in a previous study [8]. In this study, the replaced plasmid was used as a polymerase chain reaction (PCR) template for the amino acid substitution of 3C C142T (using 5′-CCATGGATGGAGACACCATG-3′ and 5′-CACTACAATGTCTTTGTAGGTA-3′). To replace the 3B1 and 3B2 regions with two 3B3 segments, we used the full genome cDNA vector that had 3B1 and 3B2 deleted and inserted 3B3 dimer genes amplified with specific primers (5′-AGGACCGACCACAAGCTGAAGGACCTTACGAGGGACCGGT-3′ and 5′-TCGGTCGGTGGGGCACCACTCTCAGTGACAATCAAGTTCT-3′) using synthetic 3B3 dimer genes as the PCR template in the 3B1 and 3B2 sites [9]. The P1 region (VP4, VP2, VP3, and VP1) of O_1_Manisa was replaced with P1 from A/POC/2010 (GenBank No. KC588943.1). The VP1 region was replaced with VP1 from A22 Iraq (GenBank No. AY593763.1) to generate “Apo22”. In addition, “Apo22-B” was produced by inserting amino acids 140–160 from VP1 of A/BAN/2013 (GenBank No. KJ754939.1; G-VII) into VP1 using the below primers and Phusion High-Fidelity DNA Polymerase (Thermo Fisher Scientific, Vantaa, Finland), according to the procedure of the manufacturer. The recombinant plasmids were sequenced by Macrogen Corporation (Geumcheon-gu, Seoul, Republic of Korea) to confirm their amino acid substitutions.

Apo22-B F; 5′-TCGCGGCGCGAGTCGCTGCTCAACTCCCTGCCGCGAGGGTC-

        GCCGCTCA-3′ and

Apo22-B R; 5′-GCTGTCCCAGGTCACCCCGTACACGCCCACTCGCGAGAGGC-

        CCTAGGTC-3′ cloning.

### 2.3. Virus Recovery and Cell Culture

The recovery of viruses was carried out using the same method as in a previous study [10]. Briefly, the recombinant cDNA plasmids were linearized by using the restriction enzyme SpeI (NEB, Ipswich, MA, USA), and the BHK/T7-9 cells, cells expressing T7 RNA polymerase, were transfected with infectious cDNA using lipofectamine 3000 (Invitrogen, Waltham, MA, USA). After incubation at 37 °C in a 5% CO_2_ atmosphere for 72 h, the viruses were harvested through three freezing and thawing cycles. The chimeric viruses were then used for the infection of fresh ZZ-R 127 cells and then BHK-21 cells were infected. To differentiate the wild-type FMDV from the chimeric viruses, structural proteins (SPs) and nonstructural proteins (NSPs) were confirmed using an FMDV Rapid kit (Princeton Bio Meditech Corporation, Princeton, NJ, USA). The virus serotype was confirmed using a VDRG FMDV 3Diff/PAN Ag Rapid Kit (MEDIAN Diagnostics, Chuncheon-si, Republic of Korea).

### 2.4. Virus Replication and Purification

Suspended BHK-21 cells were infected with viruses and incubated for 16 h in a shaking incubator. The observation continued until the complete cytopathic effect (CPE) was detected. The propagated virus was harvested by a process of freezing and thawing. Cell debris was removed by centrifugation at 4000 rpm at 4 °C for 20 min. The viruses were then inactivated using 0.003N binary ethylenimine (BEI; Sigma-Aldrich, St. Louis, MO, USA) reagent in a shaking incubator at 75 rpm at 26 °C for 24 h. The residual BEI was neutralized by the addition of 10% volume of 1M sodium thiosulfate (Daejung Chemicals, Siheung-si, Republic of Korea) to a final concentration of 2%. To confirm the presence of the viable virus, the supernatants that were treated with BEI and neutralized were passaged over twice to ZZ-R and BHK-21 cells. The supernatant was confirmed to be completely inactivated and then precipitated with 7.5% polyethylene glycol (PEG) 6000 (81253; Sigma-Aldrich, St. Louis, MO, USA) and 2.3% NaCl (S3014; Sigma-Aldrich, St. Louis, MO, USA) reagents overnight at 4 °C. To resuspend the pellet, Tris-NaCl buffer was added. Then for purification, the samples were centrifuged through a 15–45% sucrose gradient in Tris-NaCl buffer at 4 °C at 30,000 rpm (SW41 rotor) for 4 h. The antigen quantification was performed by spectrophotometric analysis at a wavelength of 259 nm. Purified antigens were discretely adsorbed on carbon-coated copper grids, and visualization was performed using a transmission electron microscope (TEM; Hitachi H7100FA, Tokyo, Japan).

### 2.5. Preparation of the Experimental Vaccines

The vaccine to be tested was prepared using the same procedure as described in a previous study [11]. Briefly, in order to prepare a vaccine for a single dose for pigs, 15 μg of each of the purified Apo22 and Apo22-B 146S antigens were individually mixed with ISA 206VG (Seppic, Paris, France) at a ratio of 1:1 (*v*/*v*). Then, 10% aluminum hydroxide gel (Rehyragel HPA; General Chemical, Moorestown, NJ, USA) and 0.5 μg saponin (Sigma-Aldrich, St. Louis, MO, USA) were added sequentially for the preparation of the water-in-oil-in-water formulation. The vaccine candidates tested in mice were prepared in the same manner but at lower doses.

### 2.6. Vaccination and Viral Challenge in Mice

Seven-week-old C57BL/6 female mice (KOSA-BIO, Seongnam-si, Republic of Korea) were used in this study. The mice were vaccinated 0, 1/10, 1/40, 1/160, or 1/640 of the 15 μg antigen dose vaccinated to the pigs. Seven days post-vaccination (dpv), the mice were challenged with a virus (1 × 10^5^ TCID_50_/0.1 mL injected intraperitoneally) and observed for seven days.

### 2.7. Vaccination and Viral Challenge in Pigs

The antigen was mixed with ISA 206 adjuvant, aluminum hydroxide gel, and saponin. The pigs (*n* = 12) were vaccinated with a single dose of 15 μg/mL of antigen. Blood was collected at −28, −21, −14, −7, 0, 2, 4, 6, and 8 (optional up to 12) days post-challenge (dpc), and the pigs were challenged with the A/POC/2010 (*n* = 6), A/GP/2018 (*n* = 6), and A/NEP/2017 (*n* = 6) viruses (each at 1 × 10^5^ TCID_50_/0.1 mL) at 28 dpv on their footpad. The groups of pigs were separately reared and isolated when they observed clinical signs of FMD after the virus challenge. Oral swabs and serum samples were taken up to 8 or 12 dpc. Blood samples were collected every two days. The oral swabs were taken every day using the BD Universal Viral Transport Kit (BD, Becton, Dickinson and Company, Franklin Lakes, NJ, USA). Clinical symptoms were observed every day post-challenge. The clinical symptom scores were calculated according to the following criteria (maximum: 15 points): (a) body temperature of 40 °C (1 point), >40.5 °C (2 points), or >41 °C (3 points); (b) lameness (1 point); (c) hoof and foot vesicles (1–2 points per foot); and (d) snout, lip, and tongue vesicles (1 point for each affected area).

### 2.8. The Immunogenicity of the Candidate Vaccine in Pigs

The 8- to 10-week-old pigs (*n* = 5) were vaccinated with 15 μg/mL antigen. The antigen was mixed with ISA 206 adjuvant, aluminum hydroxide gel, and saponin. The sera of the pigs were taken at 0, 14, 21, 28, 42, 56, and 84 dpv. The pigs were boosted at 28 dpv.

### 2.9. Detection of Antibodies against Structural Proteins

Serum antibodies against FMDV SP were detected using the VDPro FMDV A Ab ELISA (MEDIAN Diagnostics, Chuncheon, Republic of Korea). The animal was considered to have exhibited an immune response when the percent inhibition OD value of the sample ≥ 0.4 according to the manufacturer’s instructions.

### 2.10. Virus-Neutralization Test (VNT)

Serum samples obtained from pigs after vaccination were inactivated in a constant temperature water bath at 56 °C for 30 min. Serum samples were inactivated and then incubated with FMDV (100 TCID_50_) for 1 h, followed by the addition of LFBK cells and incubation for 3 d. To identify the virus-neutralization (VN) titer, the CPE was checked, which was calculated as the log10 of the reciprocal serum dilution required to neutralize 100 TCID_50_ of the virus. The A/POC/2010, A22 Iraq, A/GP/2018, and A/NEP/2017 viruses were used in the VNT.

### 2.11. Virus Detection in Vaccinated and Challenged Pigs

Real-time PCR was performed on sera and swabs from the experimental animals. Swab samples were collected from the mouth using cotton swabs. To extract total cellular RNA, QIAcube HT (QIAGEN, Hilden, Germany) was used according to the manufacturer’s instructions. qRT-PCR was carried out using the one-step prime-script RT-PCR kit (Bioneer, Daejeon, Republic of Korea) according to the manufacturer’s protocol. The primers used in the FMDV 3D region are as follows.: sense 5′-GGAACYGGGTTTTAYAAACCTGTRAT-3′ and antisense 5′-CCTCTCCTTTGCACGCCGTGGGA-3′. The probe was 5′-CCCADCGCAGGTAAAGYGATCTGTA-3′. Its 5′ end was labelled with 6-FAM and the 3′ end with TAMRA. The CFX96 Touch Real-Time PCR Detection System (Bio-Rad, Hercules, CA, USA) was used to perform virus quantification.

### 2.12. Statistical Analysis

The statistical relationships were decided between the experimental groups and the negative control groups. *t*-tests were carried out using GraphPad Prism (ver. 5.0; GraphPad Software, San Diego, CA, USA) and GraphPad Instant (ver. 3.05; GraphPad Software). Data are shown as the mean ± standard deviation. Statistical differences were regarded as significant if * *p* < 0.05, ** *p* < 0.01 and *** *p* < 0.001.

### 2.13. Ethics Statement

The animal experiments were carried out in stringent accordance with the recommendations of the Animal and Plant Quarantine Agency (APQA)’s guide for the care and use of laboratory animals. All animal procedures had been approved by the Institutional Animal Care and Use Committee (IACUC) of the APQA, Republic of Korea (approval no. 2019-461). All efforts were strived to minimize animal suffering. 

## 3. Results

### 3.1. Construction of Chimeric Viruses

**Figure 1:**
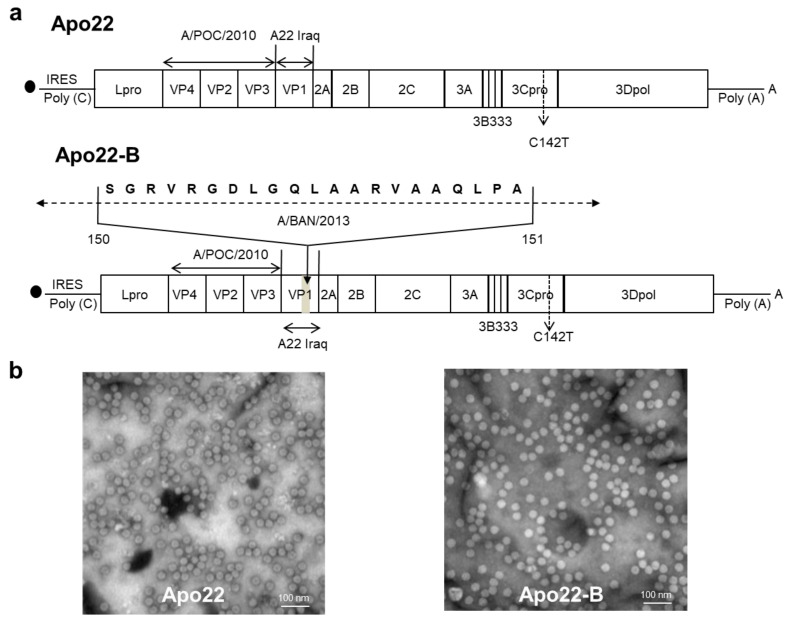
Construction of the chimeric foot-and-mouth disease viruses Apo22 and Apo22-B. (**a**) Schematic depiction of the structural proteins of the chimeric Apo22 and Apo22-B viruses. (**b**) Electron microscopy images of representative chimeric virus particles. Bar: 100 nm.

The full genome cDNA of the O_1_Manisa (AY593823.1) was cloned under the T7 RNA polymerase binding site of the pBluescript SKII vector. VP4, VP2, and VP3 from A/POC/2010 (KC588943.1) and VP1 from A22 Iraq (AY593763.1) were inserted into the host strain to prepare the Apo22 virus. That is, Apo22 expressed VP4, VP2, and VP3 from A/POC/2010 and VP1 from A22 Iraq. To prepare the Apo22-B virus, residues 140–160 from A/BAN/2013 (KJ754939.1) VP1 were added to Apo22 (Figure 1a). The viral particles were recovered from cDNA-transfected cells. As in previous studies [11], the 3B1B2 segment in the viral genome was replaced with two 3B3 segments, and the chimeric and wild-type viruses were distinguished using a simple antigen diagnostic kit (lateral flow device) capable of detecting NSP through the lack of reaction at the replaced site. In addition, electron microscopy was used to confirm that the chimeric viruses were 25 nm in diameter and thus similar in size to wide-type viruses (Figure 1b).

### 3.2. The Apo22 and Apo22-B Vaccines Protected Mice against Challenge with Type A Viruses

The protective properties of the candidate vaccines were evaluated in mice. Mice were vaccinated with the dose used in the pigs: 1/10 (1.5 µg of the 146S antigen in 0.1 mL), 1/40, 1/160, and 1/640. The mice were then challenged with four type A viruses: A/POC/2010, A/GP/2018, A22 Iraq, and A/NEP/2017. All mice challenged with A/POC/2010, A/GP/2018, and A22 Iraq survived. When mice were challenged with A/NEP/2017, only 60% and 80% of the mice that were vaccinated with the 1/640 dose of Apo22 and Apo22-B, respectively, survived. Therefore, the 50% protective dose (PD_50_) of the Apo22 and Apo22-B vaccines against A/POC/2010, A/GP/2018, and A22 Iraq in mice was determined to be >128.0 PD_50_ (1.5 µg/dose in mice), whereas against A/NEP/2017 it was determined to be 73.5 and 97.0 PD_50_, respectively (Table 1). Weight loss was only observed in some of the mice that were vaccinated with the 1/640 dose of the Apo22 and Apo22-B vaccines and were challenged with A/NEP/2017. In contrast, no weight loss was observed in any of the groups vaccinated with the Apo22 and Apo22-B vaccines and challenged with A/POC/2010, A/GP/2018, and A22 Iraq (Supplementary Figures S1 and S2). Hence, of the two candidate vaccines studied, the Apo22-B vaccine showed better protection against A/NEP/2017 than the Apo22 vaccine.

### 3.3. The Apo22-B Vaccine Completely Protected Pigs against Challenge with Type A Viruses

**Figure 2:**
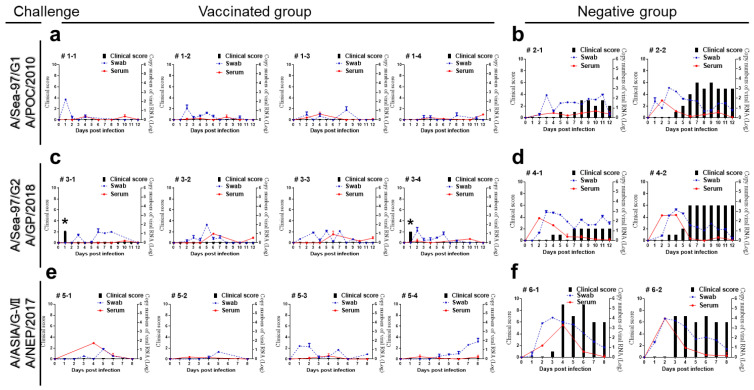
The clinical scores, viremia, and virus excretion recorded in vaccinated and negative group pigs challenged with either A/POC/2010, A/GP/2018, or A/NEP/2017. (**a**) Vaccinated group pigs (*n* = 4) after A/POC/2010 virus challenge. (**b**) Negative group pigs (*n* = 2) after A/POC/2010 virus challenge. (**c**) Vaccinated group pigs (*n* = 4) after A/GP/2018 virus challenge. (**d**) Negative group pigs (*n* = 2) after the A/GP/2018 virus challenge. (**e**) Vaccinated group pigs (*n* = 4) after A/NEP/2017 virus challenge. (**f**) Negative group pigs (*n* = 2) after the A/NEP/2017 virus challenge. * Temporary increase in body temperature. Clinical scores (black bar), viremia (blue, oral swab and red, serum).

Pigs were challenged with type A viruses on their footpad four weeks after vaccination with the Apo22-B vaccine (Table 2). After the challenge, the clinical score in the negative group remained higher from 3 dpc until 8 or 12 days compared with the vaccinated group. Two of the four pigs that were challenged with A/GP/2018 were observed to have a temporary increase in body temperature at 1 dpc. Virus shedding was observed in all the pigs in the vaccinated group. In contrast, in the control group, mild clinical symptoms appeared at the injection site and were continuously observed from 3 dpc to the end of the 8- or 12-day observation period (Figure 2). 

### 3.4. Comparison of Virus-Neutralizing Antibody Titers in Apo22-B-Vaccinated Pigs after Challenge with Type A Viruses 

**Figure 3:**
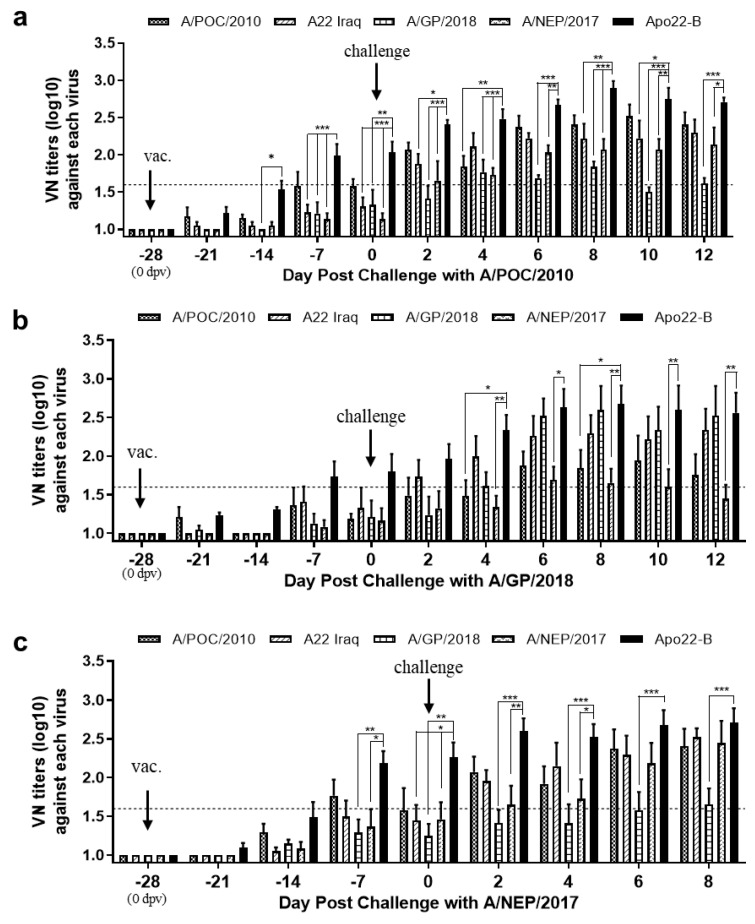
Virus-neutralization antibody levels detected in pigs that received the Apo22-B vaccine and were challenged with virus. Titers of virus-neutralizing antibodies generated in pigs challenged with A/POC/2010 ((**a**); *n* = 4), A/GP/2018 ((**b**); *n* = 4), or A/NEP/2017 ((**c**); *n* = 4). The titers are expressed as log10 values. A titer ≥ 1.6 log10 (dotted line) was considered positive. Statistical analyses were performed using two-way ANOVA followed by Turkey’s test. * *p* < 0.05; ** *p* < 0.01; and *** *p* < 0.001.

The VNT was used to quantitate and compare the neutralizing antibodies generated in pigs after the Apo22-B vaccination and were challenged with type A viruses. Four weeks after vaccination, the titers of the Apo22-B-neutralizing antibodies were similar across all the pigs: > 1.6 log10 VN titer (Figure 3). The pigs that were challenged with A/POC/2010 showed a similar increase in both their A/POC/2010- and Apo22-B-neutralizing antibodies. They had fewer A22 Iraq-neutralizing antibodies and non-significant levels of neutralizing antibodies against A/NEP/2017 and A/GP/2018 (Figure 3a). The pigs that were challenged with A/GP/2018 demonstrated an increase in neutralizing antibodies against A/GP/2018 that was similar to the increase observed in neutralizing antibodies against Apo22-B. They had fewer neutralizing antibodies against A22 Iraq, followed by those against A/POC/2010, and non-significant levels of A/NEP/2017-neutralizing antibodies (Figure 3b). The pigs that were challenged with A/NEP/2017 showed a similar increase in both their A/NEP/2017- and Apo22-B-neutralizing antibodies. They had relatively higher levels of neutralizing antibodies against A22 Iraq, followed by those against A/NEP/2017 and A/POC/2010. The titers of A/GP/2018-neutralizing antibodies in these animals were not significant (Figure 3c).

### 3.5. Comparison of Virus-Neutralizing Antibody Titers in Vaccinated Pigs

**Figure 4:**
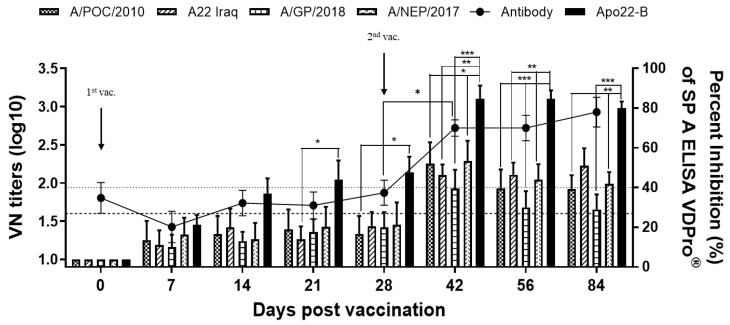
Serological responses to foot-and-mouth disease viruses in farm pigs vaccinated with Apo22-B. Quantitation of antibodies specific for structural proteins and with virus-neutralizing activity against various type A viruses present in farm pigs that received the Apo22-B vaccine (*n* = 5). A titer ≥ 1.6 log10 (dotted line) was considered positive. Antibodies specific to structural proteins were measured by ELISA. Statistical analyses were performed using two-way ANOVA followed by Turkey’s test. * *p* < 0.05; ** *p* < 0.01; and *** *p* < 0.001.

The antibodies titer and virus-neutralizing antibodies present in vaccinated farm pigs were quantitated and compared. A VNT was conducted to analyze the ability of generated antibodies to neutralize various type A viruses that share a genetic lineage with the chimeric vaccine strain used in the candidate vaccine. It was found that the farm pigs had elevated antibody titers (>1:100) a mere three weeks after vaccination that gradually increased over four weeks. The titers of the neutralizing antibodies against A/POC/2010, A22 Iraq, A/GP/2018, and A/NEP/2017 were not high. However, the antibodies that neutralized the various type A viruses that were related to the vaccine strain increased in titer after the second vaccination. Notably, the A22 Iraq- and A/NEP/2017-neutralizing antibody titers were significantly elevated, as shown in Figure 4. 

## 4. Discussion

The antigenic variation found within FMDVs is a distinguishing characteristic of RNA viruses. This is the mechanism by which infectious pathogens modify their surface proteins to avoid detection by the host’s immune system. It involves genetic mutations that result in the substitution of amino acids [12]. Thus, immune recognition of RNA viruses can be complicated by rapid mutation within progeny virus particles during infection. In terms of FMDV, RNA polymerase, which has low efficiency and is prone to errors, can induce mutations that affect immunologically linked antigenic determinants [13], and type A viruses are the most genetically and antigenically diverse, making them difficult to control with vaccines [14].

In Asia, type A viruses are less prevalent than type O viruses. However, they continue to occur [15]. Type A viruses are antigenically diverse; more than 32 genotypes have been reported [16]. Therefore, there are numerous vaccine strains compared to type O vaccine strains. In the Middle East, the A22 Iraq/24/64, A-Iran-96, A-Iran-99, A-Iran-05, and A/TUR/2006 vaccine strains have been used [17]. In Argentina, A24 Cruzeiro and A/ARG/2001 have been used [18]. In the East African region, three AFRICA topotype lineages are present (I, IV, VII), which are A-ERI-1998, A-ETH-06-2000, and A-KEN-05-1980, and one ASIA topotype (A-Iran-05) [19]. The A/IND/40/2000 vaccine strain is in use in India [3]. In Korea, Merial Animal Health’s A22 Iraq/24/64, FGBI ARRIAH’s A Zabaikalsky, Biogenesis Bago’s A24 Cruzeiro, and A2001 Argentina vaccine strains are used [20].

Although the A/ASIA/G-VII virus has not yet emerged in Korea, it occurs frequently in neighboring countries; thus, there is a significant risk of not only A/ASIA/Sea-97 G1 and A/ASIA/Sea-97 G2 outbreaks in Korea but also A/ASIA/G-VII outbreaks [4]. For this reason, there is an urgent need to develop new vaccine strains that can provide effective protection against antigenically diverse type A FMDVs [7]. However, to date, no studies have focused on developing a vaccine strain that can protect susceptible animals from three topotype viruses. 

The chimeric vaccine strain developed in this study (Apo22-B) grew well and efficiently produced viable antigens. Despite the relatively low neutralization antibody titers observed after the first vaccination, protection was still achieved against all three topotypes. Others have also shown that vaccination with type A FMDVs can induce protection in heterologous virus challenge experiments despite low r1 values [21,22]. These findings indicate that factors other than neutralizing antibodies may be more important in establishing protection against type A FMDVs. For example, in vaccinated animals with low levels of neutralizing antibodies, cytokines (e.g., IFN-γ), other cellular mechanisms, and non-neutralizing antibodies may all play roles in protection [23,24,25], particularly against heterologous virus challenge. 

It is likely that there will continue to be type A FMDV outbreaks around the world in the future, and to counter the emergence of new mutant strains, multiple vaccine development strategies are required. The findings of this study provide critical insights that can be used in the ongoing worldwide development of FMD vaccines. As part of this effort, we plan to develop an FMD vaccine suitable for Asia in the near future. 

The development of novel FMD vaccine strains in this study is limited for commercialization as a vaccine. This is because it is difficult to use a large number of pigs due to IACUC. In this respect, it seems desirable to carry out a large number of animal experiments.

## 5. Conclusions

The study demonstrated that the Apo22-B vaccine candidate strain provides broad-spectrum protection against all three different type A viruses. The Apo22-B vaccine strains have proved to be a broad vaccine candidate strain for the prevention and control of various type A pandemic FMDV infections in Korea and Asia. This Apo22-B vaccine strain has been shown to provide broad protection in experimental mice and pigs. This Apo22-B vaccine strain can protect against all three different type A viruses, including A/Sea-97/G1, A/Sea-97/G2, and A/ASIA/GⅦ, making it an appropriate vaccine strain for use in regions where outbreaks of these viruses are frequent. Even if they belong to the three genotypes, it will be necessary in the future to classify viruses with different genetic characteristics and to determine exactly whether protection against them is possible.

## Figures and Tables

**Table 1:** Summary of the 50% protective dose (PD_50_) values in mice of the vaccine candidates.

Vaccine Candidates	Challenge Viruses (PD_50_)			
	G1 (A/POC/2010)	G2 (A/GP/2018)	A22 Iraq	GVII (A/NEP/2017)
Apo22	>128.0	>128.0	>128.0	73.5
Apo22-B	>128.0	>128.0	>128.0	97.0

C57BL/6 mice (*n* = 4 or 5/group) were vaccinated with 1/10, 1/40, 1/160, and 1/640 doses of the Apo22 and Apo22-B antigens. The candidate vaccines were injected intramuscularly into mice that were later challenged with type A foot-and-mouth disease viruses (1 × 10^5^ TCID_50_/0.1 mL of A/POC/2010, A/GP/2018, A22 Iraq, or A/NEP/2017). The survival rates and body weights of the mice were monitored for seven days post-challenge (data are shown in Supplementary Figures S1 and S2).

**Table 2:** Experimental designs of the Apo22-B vaccine efficacy study.

Group (Cage)	No. of Animal	Experimental Group	Challenge Virus (1 × 10^5^ TCID_50_/0.1 mL)	Days of Blood Collection (dpc)	Days of Oral Swab Collection (dpc)	Comments
1 (1)	4	Apo22-B	A/POC/2010	−28, −21, −14, −7, 0, 2, 4, 6, 8, 10, 12	0, 1, 2, 3, 4, 5, 6, 7, 8, 9, 10, 11, 12	If symptoms were observed after the challenge, they were isolated in empty cage.
2 (1)	2	Negative group				
3 (2)	4	Apo22-B	A/GP/2018			
4 (2)	2	Negative group				
5 (3)	4	Apo22-B	A/NEP/2017	−28, −21, −14, −7, 0, 2, 4, 6, 8	0, 1, 2, 3, 4, 5, 6, 7, 8	
6 (3)	2	Negative group				

## Data Availability

Data are contained within the article.

## Supplementary Materials

The following supporting information can be downloaded at https://www.mdpi.com/article/10.3390/vaccines12010064/s1, Figure S1: Determination of 50% protective dose against virus challenge in the C57BL/6 mice immunized with Apo22 vaccine.; Figure S2: Determination of 50% protective dose against virus challenge in the C57BL/6 mice immunized with Apo22-B.
