# Supplementary material for: Evaluation of a Vaccine Candidate Designed for Broad-Spectrum Protection against Type A Foot-and-Mouth Disease in Asia
Source: Vaccines (Basel). 2024 Jan 9;12(1):64. doi: 10.3390/vaccines12010064 (PMC10819240; doi:10.3390/vaccines12010064)
Supplement: Supplementary file 1 [file vaccines-12-00064-s001.zip › vaccines-2796662-supplementary.pdf]

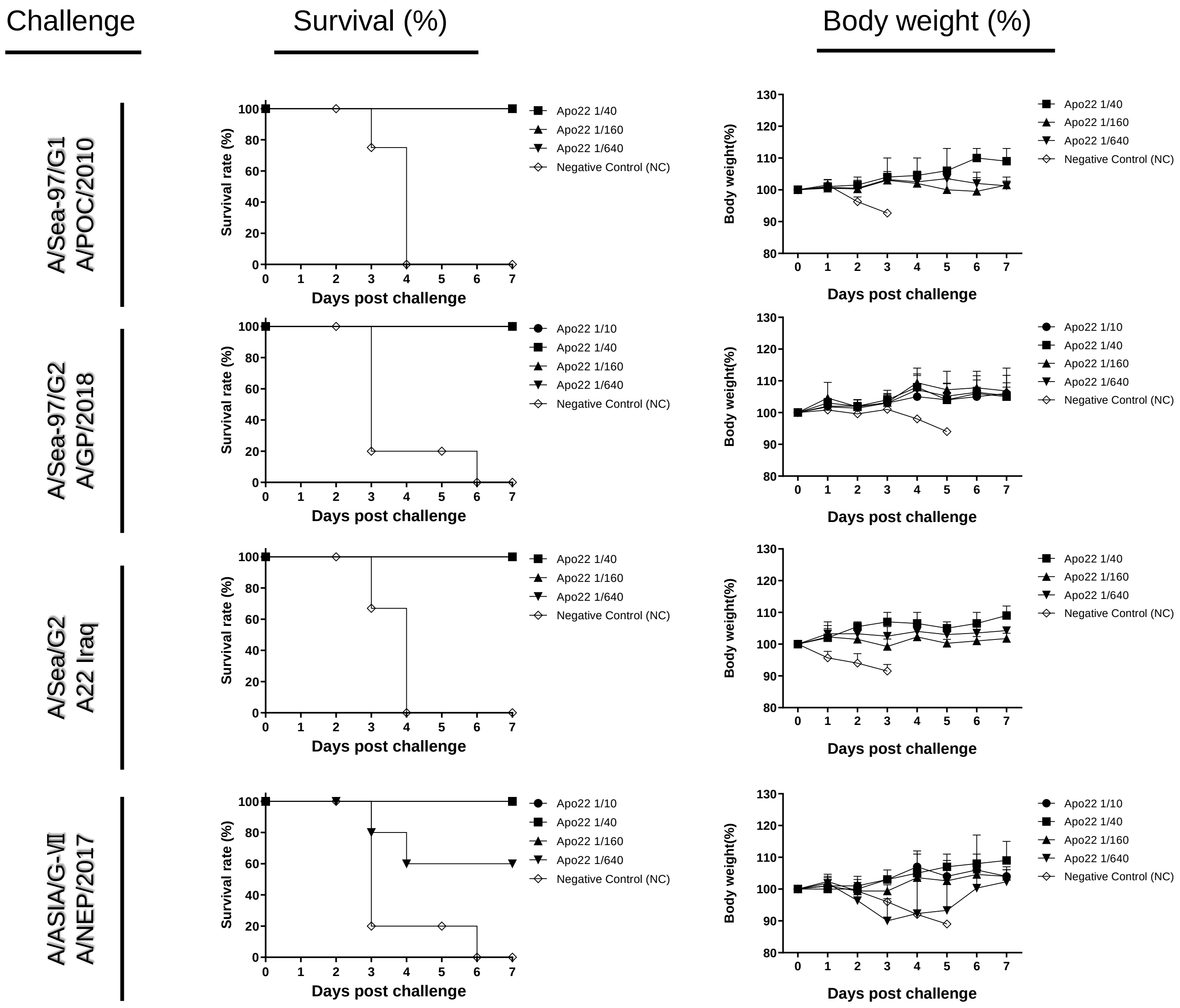

Supplementary Figure 1 : Determination of 50% protective dose against virus challenge in the C57BL/6 mice immunized with Apo22 vaccine.
The survival rate and change of body weight with 1 × 10^5 $TCID_{50}$/0.1 mL of A/POC/2010, A/GP/2018 or A/NEP/2017.

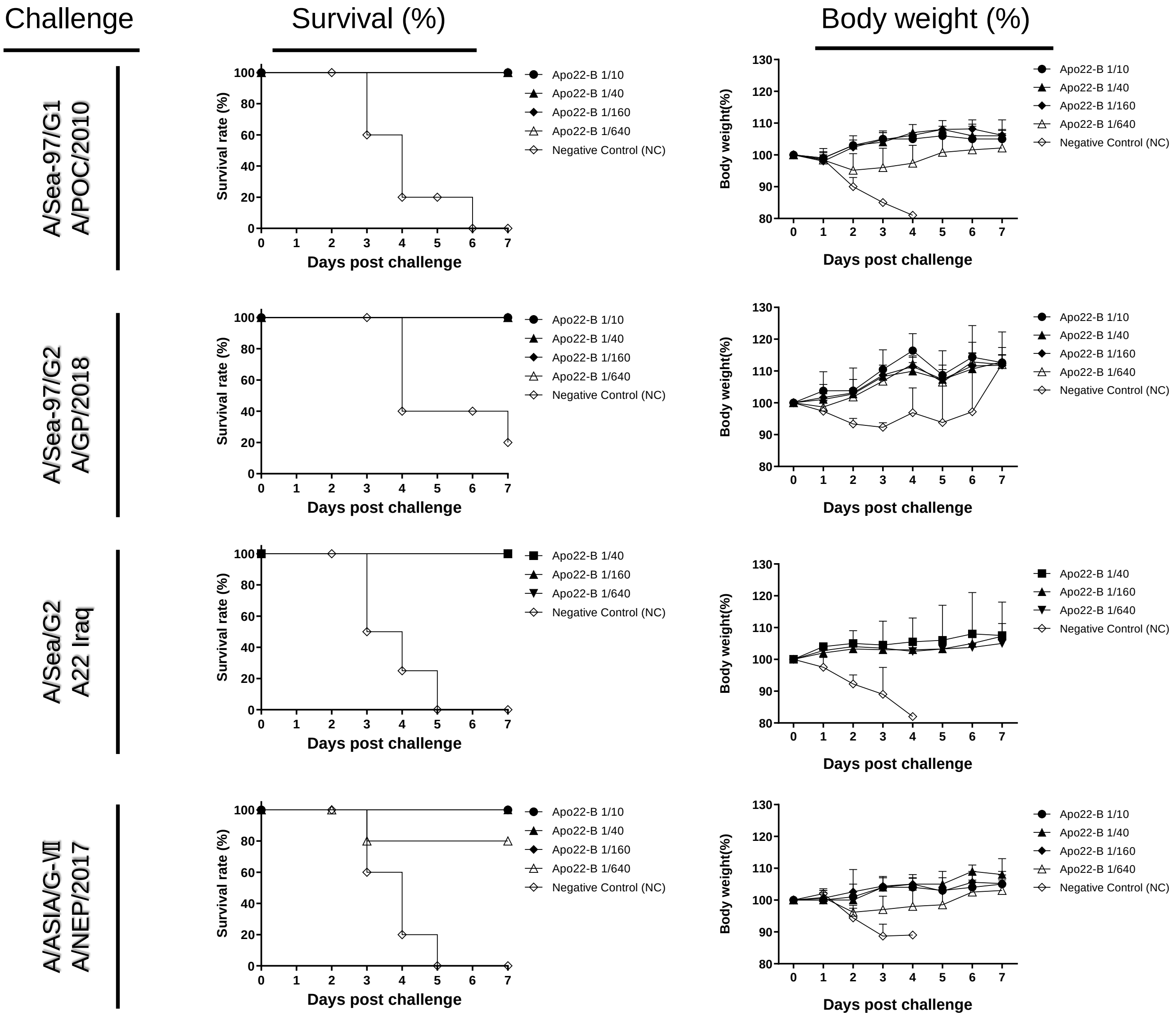

Supplementary Figure 2 : Determination of 50% protective dose against virus challenge in the C57BL/6 mice immunized with Apo22-B vaccine.
The survival rate and change of body weight with 1×10^5 $TCID_{50}$/0.1 mL of A/POC/2010, A/GP/2018 or A/NEP/2017.
